# The role of normative beliefs in the mediation of a school-based drug prevention program: A secondary analysis of the #Tamojunto cluster-randomized trial

**DOI:** 10.1371/journal.pone.0208072

**Published:** 2019-01-07

**Authors:** Zila M. Sanchez, Juliana Y. Valente, Thiago M. Fidalgo, Ana Paula Leal, Pollyanna Fausta de Pimentel de Medeiros, Hugo Cogo-Moreira

**Affiliations:** 1 Department of Preventive Medicine, Universidade Federal São Paulo, São Paulo, Brazil; 2 Department of Psychiatry, Universidade Federal São Paulo, São Paulo, Brazil; 3 Department of Psychobiology, Universidade Federal São Paulo, São Paulo, Brazil; 4 Therapeutic Residency Coordination, Instituto de Medicina Integral Profº Fernando Figueira, Recife–PE, Brazil; 5 Division of Methods and Evaluation, Department of Education and Psychology, Freie Universität Berlin, Berlin, Germany; TNO, NETHERLANDS

## Abstract

**Aims:**

To investigate the mediating effects of normative beliefs of drug use on the effects of the #Tamojunto school-based prevention program (*Unplugged*).

**Design:**

Secondary analysis of a cluster randomized controlled trial.

**Setting:**

Brazil. Participants: A total of 6,391 adolescents (12.68 y.o) from 72 public schools in 6 Brazilian cities. Intervention: Schools were assigned to an experimental condition (#Tamojunto curriculum) or a control condition (no prevention program). Measurements: Baseline data were collected prior to program implementation, and follow-up data were collected 9 and 21 months later. The substances examined were alcohol (including binge drinking), tobacco, marijuana and inhalants. Five in-parallel mediation models evaluated whether the positive and negative beliefs were mediators of the likely effects of the intervention on drug use.

**Findings:**

Lack of evidences regarding differences in normative beliefs or drug use were found between the intervention and control groups. However, there was a clear association between negative drug beliefs and lower consumption (i.e. OR = 0.78; 95% CI 0.70; 0.87, for cannabis use) as well as between positive drug beliefs and higher consumption (i.e. OR = 1.77; 95% CI 1.56; 2.02, for cannabis use) independent of the assigned group.

**Conclusions:**

These results suggest that there is a lack of evidence that the program impact the normative beliefs, as proposed by the theoretical model of the program, suggesting that modifications are needed to produce the intended effect of the program. Negative normative beliefs seem to be a potential protective factor for drug use, but the program’s effect itself on drug use via normative beliefs was not found to be statistically significant. Program activities intended to affect normative beliefs should be improved.

## Introduction

Since the 1980s, prevention programs have been created to address the major public health [1,2] issue of the premature use of substances during adolescence [3]. Programs based on a social influence approach, which aims to strengthen personal and interpersonal skills through changes in normative beliefs, are more likely to be effective than programs based on other models [4–7].

In Brazil, although licit and illicit drug use is initiated in early adolescence, between 12 and 14 years of age [8,9], evidence-based drug use prevention programs are not typically implemented in schools [10]. In 2013, to address this gap, the Brazilian government conducted a transcultural adaptation process and implemented the *Unplugged* program as a public policy. *Unplugged* is based on drug information, normative beliefs and life skills, showing positive results in reducing episodes of drunkenness as well as reports of frequent cannabis [11], tobacco, and any drug use [12] among European adolescents.

Unplugged was renamed #Tamojunto in Brazil and was submitted to a randomized controlled trial to evaluate its effectiveness among Brazilian students. This study showed that the program seemed to increase first alcohol use (aRR = 1.30, 95% CI 1.13–1.49) and decrease first inhalant use (aRR = 0.78, 95% CI 0.63–0.96) in the intervention group compared to the control group at the 9-month follow up [13]. Considering these contradictory results, an understanding of the mechanisms underlying the success and failure of this program is needed to identify whether the intervention is effective for changing normative beliefs and to determine where program curricula may be improved to achieve the expected outcome. In other words, does the prevention program affect the mediating variables that are targeted by the intervention as proposed in its logic model [14,15], which in turns, change the drug use as main outcome?

Normative beliefs can be defined as the perceptions of others’ approval or disapproval of some behavior [16]. Questions concerning whether substance use can be a source of pleasure or whether people who use a substance should be regretful of that are examples of inquires related to normative beliefs [17]. In a social and cultural environment with positive beliefs about alcohol, children and adolescents are more prone to use it, as they perceive this behavior as social acceptable. It is important to emphasize that there is no consensus on what to ask in order to measure normative beliefs. Qualitative open questions have already been used [18]. In this study, not only beliefs were included, but also questions about interviewees behaviors on specific situations, such as “If you were to move to a new organization that was not using circuit class therapy, or 7-day service would you advocate for these?” and “What would you like to tell researchers, or policy makers or managers of your organization that would improve stroke rehabilitation” [18]. Other study understood normative beliefs as closely related to attitudes, exerting a mediating role between pure beliefs and attitudes [19].

This comprehension of normative beliefs as mediators between cognitive concepts and attitudes seems to be of great importance for elaborating substance use prevention programs. They are targeted in prevention programs with the aim of reducing the effects of social influences in the drug use initiation process, engaging adolescents to think critically about substance use to change their perceptions towards drug use [5]. Normative beliefs are adopted in prevention programs due to the use of two complementary theoretical models, the theory of Reasoned Action–Attitude and the Planned Behavior theory, which are based on the concept that there is always an intention before the behavior itself. Attitudes contribute to modeling the intentions and result from balancing the perceived beneficial and dangerous outcomes of the behavior [15]. In this context, normative beliefs should work as mediators of the intervention effects [20,21], providing an insight into the mechanism [22] via two paths: prevention program activities modifying the mediators [23] and the mediators affecting the outcome measurements [24]. Mediation analysis can be described as the processes that lead to behavioral change, providing an overview of the mechanisms underlying program success or failure [25]. Reliance on the “criteria to establish mediation” logic described in the seminal work of Baron and Kenny [26] are largely no longer recommended by methodologists in the area of mediation analysis. More contemporary approaches has focused on the indirect effect of X on Y [27]. The indirect effect of X (#Tamojunto random assignment) on Y (drug use) through mediator (normative beliefs) quantifies the estimated difference in Y resulting from a one-unit change in X through a sequence of causal steps in which X affects M, which in turn affects Y. Thus, regardless of the intervention impact on drug use or impact the normative beliefs, contemporary mediation model has not postulate that both previously cited path must be statistically significant in order to deflagrate a mediated effect of X on Y via M.

A study that evaluated the short-term mediation factor involved in the effectiveness of the *Unplugged* program in Europe found that adolescents in the intervention group reduced positive attitudes toward drugs; positive beliefs about cigarettes, alcohol, and cannabis; and the normative perception of peers using tobacco and cannabis [5]. Other social-influence-based school prevention program studies have conducted mediation evaluations and corroborate these findings, showing that normative beliefs are significant mediators between the prevention program activities and adolescents’ drug use [28–30]. However, it should be noted that in the literature of school-based prevention programs, there is a lack of evaluations of mediation mechanisms [31].

Considering the important role that normative beliefs play in drug prevention programs, the purpose of this study is to investigate the mediating effects of the #Tamojunto school-based prevention program (Unplugged) in Brazil on adolescents drug use via two normative beliefs.

## Methods

### Study design

The present study was based on a secondary analysis of a two-arm school cluster randomized controlled trial (**Consort checklist in S1 Table and S2 Table**), in which schools were randomly assigned to either the intervention arm (#Tamojunto program) or to a control arm, receiving the usual education curriculum in Brazil (no prevention program), among adolescents in 72 public schools in 6 Brazilian cities (São Paulo, Distrito Federal, São Bernardo do Campo, Florianópolis, Fortaleza and Tubarão), located in 4 Brazilian states. Considering that this is an evaluation of a school intervention, we used the cluster design.

Excel’s macro [command RAND] was used to perform the randomization at the school level, and in the drawn school, all potential classrooms were invited to participate. Data were collected simultaneously in the control and intervention schools at three time points. Pre-test data were collected from February 10 to 21, 2014. The first follow-up assessment was carried out 9 months later (November 10 to 28, 2014), and the second follow-up assessment was conducted 21 months after baseline (November 9 to 28, 2015).

The RCT was registered at the Brazilian Ministry of Health Register of Clinical Trials (REBEC), under protocol number RBR-4mnv5g. The register on REBEC depends on the approval of the University Research Ethics Committee (REC). We have obtained the REC approval on November 2013 and have started the process in REBEC in early 2014. However, it took 7 months to be evaluated in REBEC. It is usually a very slow process (**S3 Table**). The authors confirm that all ongoing and related trials for this drug/intervention are registered.

All procedures in the present study were in accordance with the ethical standards of the institutional and/or national research committee and with the 1964 Helsinki Declaration and its later amendments or comparable ethical standards. This study and the consent procedure were approved by the Ethics in Research Committees at the University of São Paulo (#473.498) on November 23th 2013. Consent to participate in the study was written and obtained from the schools’ directors before randomization and from students, after randomization. All participants took part voluntarily after having given their free and informed consent based on the autonomy of adolescents guaranteed by the Brazilian Statute of the Child and Adolescent (Law No. 8069/1990). Moreover, parents were informed of the study by the directors and could recommend non-participation in data collection if they preferred. However, participation in the intervention was part of the school curriculum and was mandatory for all the students in the participating schools.

### Population and sample size

Based on the sample size calculation [32] defined to investigate recent binge drinking, the primary outcome of #Tamojunto trial, for a given power of 80%, a significance level of 5% and a difference between groups of 1.5 percentage points (i.e., from 5% to 3.5%), the necessary sample size for each study arm was calculated to be 2,835. To account for losses and for a high intraclass correlation, the sample was increased by 50% and had to draw 4,253 participants in each arm. The parameters used were based on a previously conducted pilot study and data regarding school absences of enrolled students [33].

The target population was students attending 7^th^ and 8^th^ grade (12 to 13 years of age) in the geographical areas of the cities participating in the study. In each of the participating municipalities, 4 to 30 schools were simply randomly selected (in proportion to the size of the city’s population) from all of the public middle schools in these locations (using the national registration list of schools from the Instituto Nacional de Estudos e Pesquisas Educacionais Anísio Teixeira (INEP). Using the schools selected to participate in the study, a second simple, random selection process was performed to match the control and intervention schools at a ratio of 1:1 by municipality.

**Fig 1:**
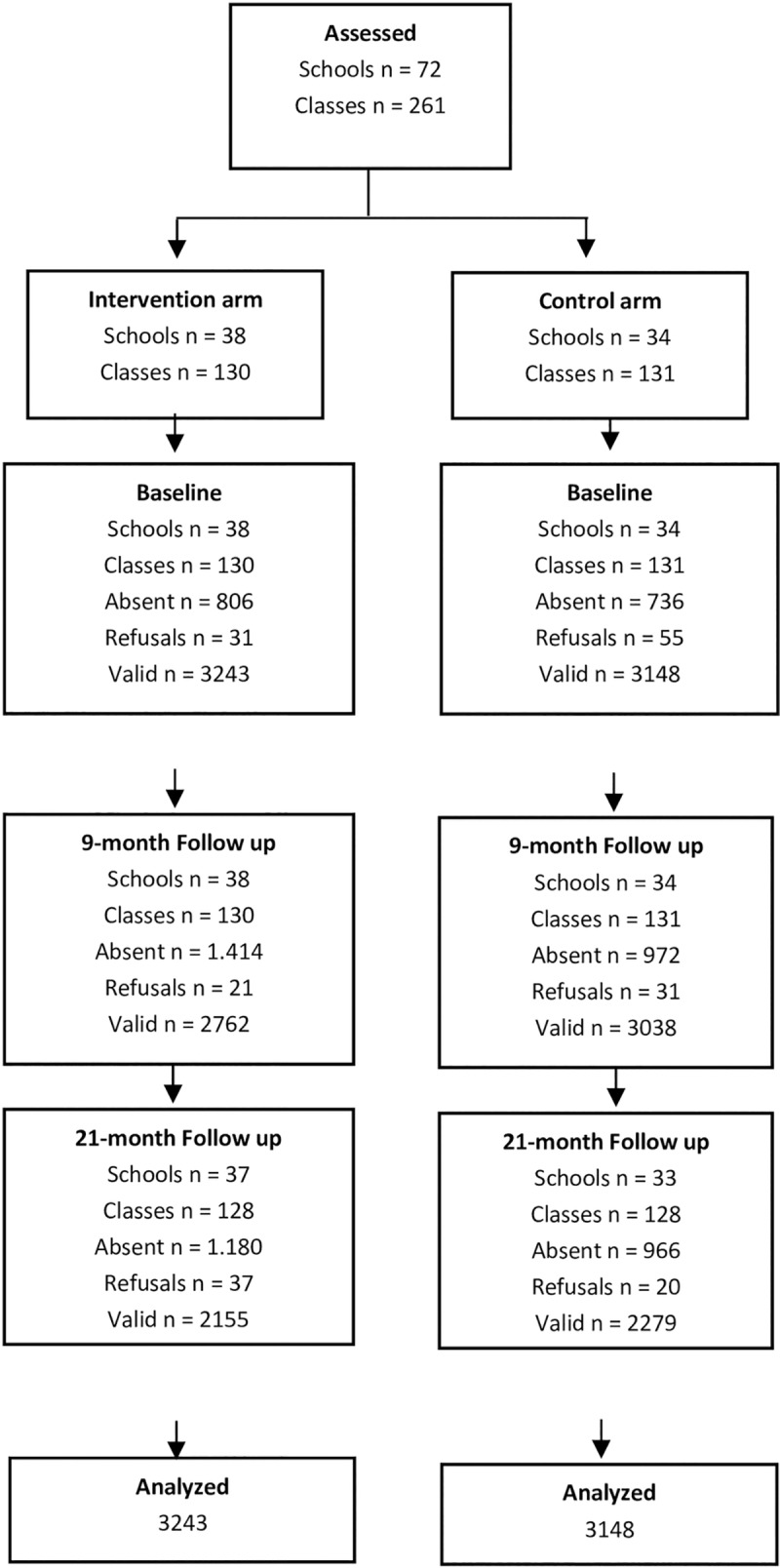
CONSORT flow diagram for the randomized controlled trial.

As each school had approximately four 8^th^ grade classes of 30 students each, at least 35 schools in the intervention arm and the same number in the control arm (total of 70 schools) were needed to access the number of students required to maintain the power of the test. Considering a 10% rate of refusal of schools, 38 schools were enrolled in each arm. A total of 72 schools accepted our invitation to participate in the study, as described in **Fig 1**. In each of the participating municipalities, 4 to 30 schools were randomly selected (in proportion to the size of the city’s population).

In each of the schools, all 8^th^ grade classes were invited to participate in the study, prior to randomization of groups. In Fortaleza, Santa Catarina and Tubarão, the 7^th^ grade classes of the selected schools were also included because these cities were in the process of changing the age of students assigned to each grade, and the State Education Secretariat requested the inclusion of the 7^th^ grade classes in the study. Details on the study design and sampling methods have been previously presented [13].

### Intervention

The *Unplugged* program was first designed by the EU-DAP group [34] and consists of 12 classes based on a social influence curriculum (4 one-hour classes on attitudes and knowledge of drugs, 4 classes on social and interpersonal skills, and 4 classes on personal skills), with an average class time of 50 minutes. The classes are delivered by class teachers trained and guided by the students’ and the teacher’s manuals. Both manuals are open-access and made available in several languages on the website www.eudap.net.

The implementation and cultural adaptation of the program were the responsibility of the Brazilian Ministry of Health (BMH) team under the supervision of the European developers (in 2013), and the evaluation was conducted by an independent team of federal university professors.

The teachers that delivered the program attended a 16-hour training program facilitated by coaches trained by the European developers, i.e., the master-trainers of the EU-DAP Intervention Planning Group [12]. To guarantee fidelity and dose, teachers were supervised monthly by the coaches from the BMH who had facilitated the initial training. At the end of each class, teachers had to complete a fidelity questionnaire to assess the dose of the program delivered. A total of 87% of the schools completed the 12 program lessons. The other 13% terminated the program between lessons 4 and 11 for two main reasons: the teachers went on medical leave, or they were not comfortable implementing the program.

The English version of the *Unplugged* material was translated into Portuguese, retaining the original format and subject (educational strategies provided in 12 classes and 3 parent workshops) but with adapted activities. Given the epidemiological profile of illegal drug use among students in Brazil, all information on heroin was excluded and replaced with information on crack-cocaine [8]. Nevertheless, the main changes were made to align the activities of the program with the Alcohol and Other Drugs Policy paradigm advocated by the Brazilian government [35], which is against the “War on Drugs” model. More details about the cultural adaptation process are described in [36].

### Instrument and variables

The instrument used for data collection was developed and tested by the EU-DAP and used in previous studies of *Unplugged* effectiveness [37]. In Brazil, we used a translated and adapted version of the EU-DAP questionnaire in Portuguese [38] that had some questions replaced with items from two questionnaires widely used in several studies among Brazilian students: a questionnaire by the World Health Organization for drug surveys at schools that was adapted by the Brazilian Center for Psychotropic Drug Information [8] and a questionnaire by PENSE (the Brazilian National Survey of School Health) that was used by the BMH [39].

The outcomes analyzed were adolescents’ past 9- and 12-month use (use in the year = yes vs. no) of the following drugs: alcohol (including binge drinking, or the consumption of five or more alcoholic drinks on a single occasion), tobacco, marijuana, and inhalants. The adjustment variables were sex, age and socio-economic class (SES) assessed using the ABEP scale [40]. To evaluate normative beliefs about drugs, we used the scale developed by EU-DAP [5] about negative and positive beliefs about drugs. This is the module from the EU-DAP questionnaire that effectively evaluate the concept of normative beliefs, rather than only attitudes. The 11-item scale assessing attitudes and beliefs about drugs contains 6 items to which a response of “Agree” would constitute a “drug-negative” response, and 5 items to which a response of “Agree” would indicate a “drug-positive” response. The positive beliefs (agree or disagree) related to drug use were as follows: “Using drugs can be a pleasant activity”, “Many things are much riskier than trying drugs”, “Using drugs is fun”, “Drugs help people to experience life fully”, and “The police should not be annoying young people who are trying drugs”. The negative beliefs (agree or disagree) related to drug use were as follows: “A young person should never use drugs”, “Everyone who tries drugs eventually regrets it”, “To experiment with drugs is to give away control of your life”, “Schools should teach the real hazards of taking drugs”, “Drug use is one of the biggest evils in the country”, “The laws about drugs should be made stronger”, and “A young person should never try drugs”.

Contrary to some studies [5] in which the indirect trajectories were estimated using single items as mediators generating multiple mediation models (also called parallel mediation models), we opted to create two parceling scores: one related to the 5 positive items and the other to the 6 negative beliefs. The parceling procedure was adopted to reduce the number of comparisons across the mediators (consequently generating a more parsimonious model). Parceling is supported by the psychometric principles based on the Principle of Aggregation [41,42] and the Law of Large Numbers [43,44]; for additional details, see [45].

To pair (link) the questionnaires of each subject at the three data collection time points (baseline and the two follow-up time points), students filled in a secret code created from their personal information. These codes protected the participants, offering anonymity and confidentiality, and at the same time allowed researchers to link the individual questionnaires collected at the different time points of the study [46]. The secret codes were matched using the Levenshtein algorithm, which identifies similarities among a set of characters. School and class codes were included in the matching process [47]. Final data is presented in S2 Appendix.

### Statistical analysis

Five in-parallel mediation models were evaluated to determine if the positive and negative beliefs were mediators of the likely effects of the group intervention assignment (random) on five different outcomes related to drug use: alcohol use, alcohol binge drinking, cannabis use, cigarette use, and inhalant use. In other words, we tested if the random assignment to the intervention (an antecedent variable) influenced the consequent variables (the five outcomes regarding drug use) indirectly through two types of beliefs, taking the school (second level) as the cluster indicator. The covariates were age, sex, SES, and the outcomes at the baseline assessment. Normative beliefs included in the analysis referred to the first follow-up data collection point (9 months after baseline). All analysis included the 72 clusters, which were analyzed via the Mplus’ COMPLEX command to deal with non-independence of the observation (i.e., children nested in schools).

**Fig 2:**
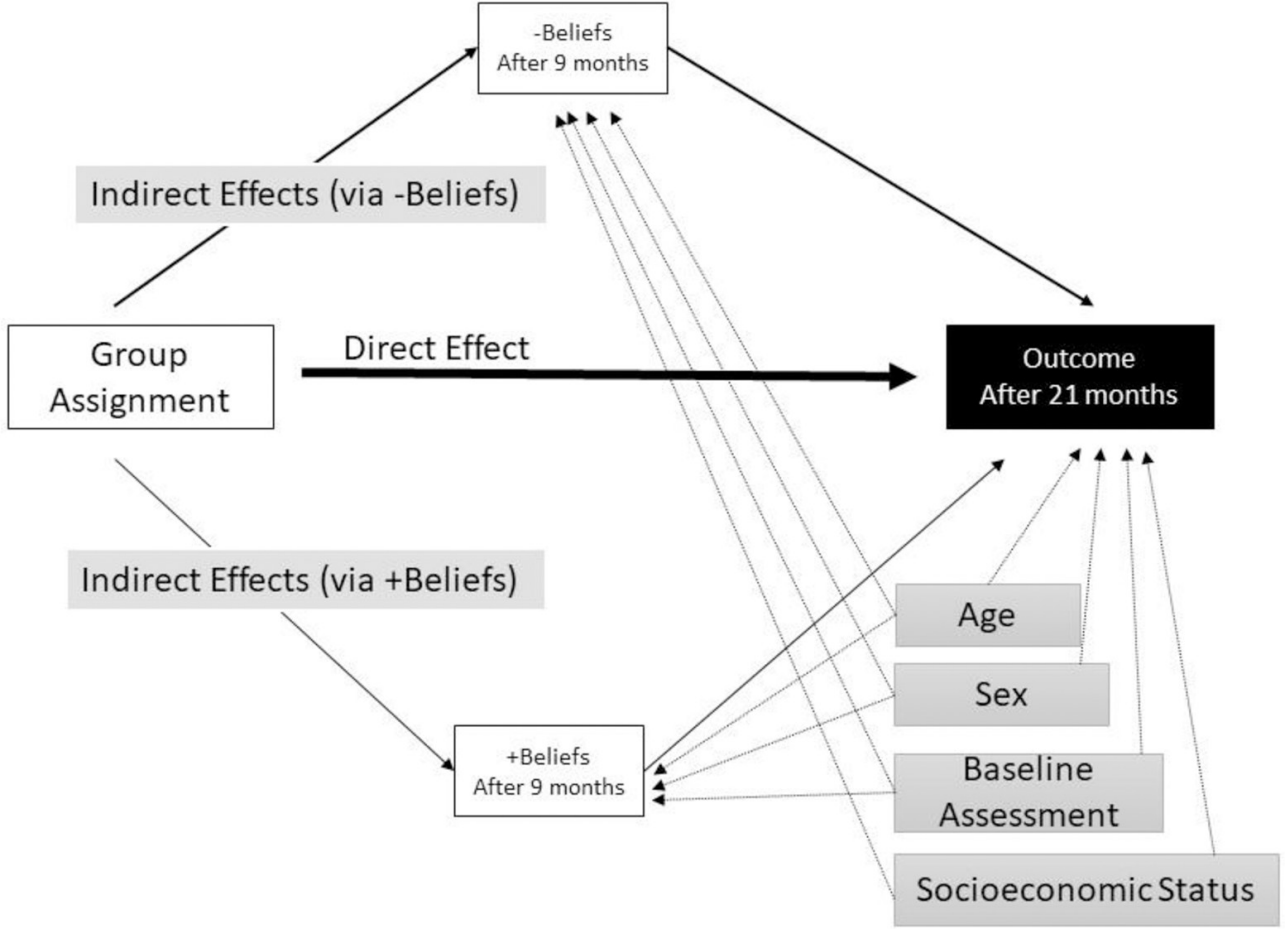
Conceptual model of the mediation model tested.

**Fig 2** shows the in-parallel mediation model. It should be noted that the covariates (in gray) were also regressed at the same time on the two mediators and on the outcomes [48].

Due to the study design, an in-cluster randomized clinical trial, missing data across the follow-up time points were imputed to fulfill the intention-to-treat (ITT) paradigm following the CONSORT statements [49]. Multiple imputations were carried out using Bayes estimation of an unrestricted variance-covariance model, which is then used to impute the missing values. Regarding the unrestricted model to use for imputation, the sequential setting available in Mplus [50] version 7.4 was selected because there is a combination of continuous and categorical variables in our mediation models. The sequential setting uses a sequential regression method, also referred to as the chained equations algorithm [51].

Multiple imputations are random draws from the posterior distribution of the missing values [52,53]. Major details regarding the multiple imputation methods can be found in [54]. Five imputation datasets were generated and used in the subsequent analyses using the method from [53] via a robust maximum likelihood (ML) estimator. We opted for such an estimator due to our complex design, where the 3,691 children are nested in 72 schools, producing an ML with odds ratios that are more comprehensible than a probit scale. To evaluate the robustness of the multiple imputation followed by ML analysis (MI-ML), we conducted a sensitivity analysis using two other analytical approaches to deal with the missing values: 1) a listwise approach, where only subjects with complete data on outcomes and covariates are analyzed; and 2) an approach using all data available to estimate the model through full information ML, where each parameter is estimated directly without first filling in missing data values for each individual, assuming values are missing at random.

Indirect effects are in logit scale, and significance was inspected by evaluating the 95% confidence intervals (when zero is contained in the interval, there is a lack of evidence for the indirect effects). The impact of the covariates, group assignment, and mediators on the outcome are expressed in odds ratios with their respective 95% confidence intervals (confidence intervals containing 1 indicate a lack of statistical significance) for the direct effect and logit for the indirect effect.

Lastly, based on the obtained indirect effect estimates described in the results section, we conducted a Monte Carlo simulation analysis to evaluate the power and other parameters related to the sample size of 6391 subjects, presented in the Supplementary File (**S3 Table**). 1,000 replications were considered and the following criteria, were we took into account to the evaluation of the adequacy of the sample size: 1) the proportion of replications for which the 95% confidence interval contains the true population parameter value, which was depicted in the first column called 95% coverage, where it is expected the values between 0.91 and 0.98. Also, in addition to this criterion, a power for the indirect effects were estimated based on the 6,391 subjects.

## Results

The sample comprised 6,391 adolescents in 72 schools (51% females, average age = 12.62 years old, standard deviation [SD] = 0.825, ranging from 11 to 15 years old). The average ABEP score was 28.02 (SD = 8.17), corresponding to a middle-class score. **Table 1** shows the frequencies of the past-year drug use at the two time points of assessment (baseline and 21 months after the intervention), together with the missing values.

**Table 1:** Descriptive statistics of past-year drug use at the two time points (baseline and 21 months after the intervention).

		Baseline			After 21 montds		
		No (valid)	Yes (valid)	Missing	No (valid)	Yes (valid)	Missing
	Alcohol Use	4329 (67.7%)	2015 (31.5%)	47 (0.7%)	1894 (29.6%)	1731 (27.1%)	2766 (43.3%)
	Alcohol Binge Drinking	5315 (83.2%)	1006 (15.7%)	70 (1.1%)	2696 (42.2%)	908 (14.2%)	2787 (43.6%)
	Cigarette Use	6058 (95.2%)	243 (3.8%)	63 (1.0%)	3353 (52.5%)	252 (3.9)	2786 (43.6%)
	Inhalant Use	5802 (90.8%)	525 (8.2%)	64 (1.0%)	3232 (50.6%)	377 (5.9%)	3609 (56.5%)
	Cannabis Use	6171 (96.6%)	156 (2.4%)	64 (1.0%)		3324 (52.0%)	276 (4.3%)	2791 (43.7%)
		Control			Unplugged		
		No (valid %)	Yes (valid %)	Missing %	No (valid %)	Yes (valid %)	Missing %
Baseline	Alcohol Use	2126 (67.5%)	1001 (31.8%)	21 (0.7%)	2203 (67.9%)	1014 (31.3%)	26 (0.8%)
	Alcohol Binge Drinking	2633 (83.6%)	487 (15.5%)	28 (0.9%)	2682 (82.7%)	519 (16.0%)	42 (1.3%)
	Cigarette Use	3005 (95.5%)	115 (3.7%)	28 (0.9%)	3080 (95%)	128 (3.9)	35 (1.1.%)
	Inhalant Use	2867 (91.1%)	254 (8.1%)	27 (0.9%)	2935 (90.5%)	271 (8.4%)	37 (1.1%)
	Cannabis Use	3050 (96.9%)	73 (2.3%)	25 (0.8%)		3121 (96.2%)	83 (2.6%)	39 (1.2%)
21 months	Alcohol Use	1005 (31.9%)	849 (27.05)	1294 (41.1%)	889 (27.4%)	882 (27.2%)	1472 (45.4%)
	Alcohol Binge Drinking	1384 (44.0%)	460 (14.6%)	1304 (41.45)	1312 (40.5%)	448 (13.8%)	1760 (54.3%)
	Cigarette Use	1724 (54.8%)	122 (3.9%)	1302 (41.4%)	1629 (50.2%)	130 (4.0%)	1484 (45.8%)
	Inhalant Use	1643 (52.2%)	202 (6.4%)	1845 (58.6%)	1589 (49.0%)	175 (5.4%)	1764 (54.4%)
	Cannabis Use	1713 (54.4%)	133 (4.2%)	1302 (41.4%)	1611 (49.7%)	143 (4.4.%)	1489 (45.9%)

The mediators, positive (mean = 1.22, SD = 1.11) and negative (mean = 4.92, SD = 1.48) beliefs, had 39.07% and 40.88% missing data points, respectively. Both the intervention and control groups showed an increase in the prevalence of all drugs evaluated during the 21 months period.

**Table 2** shows the impact of all covariates on the outcomes, through direct effects. The direct effects, which represent the simple association between the variables, showed that positive beliefs about drugs are associated with the reporting of past-year consumption for all drugs. There is a gradient of association from licit to illicit drugs, where the strongest association came from cannabis use. Students that reported cannabis use at 21 months were 77% (OR = 1.77; 95% CI 1.56; 2.02) more likely to also have reported positive drug beliefs at 9 months, independent of group allocation, sex, age, SES and baseline drug use.

**Table 2:** The direct effect and covariate effects on the use of the five drugs examined.

	Group	Age	Sex	SES	Baseline	Positive Beliefs	Negative Beliefs
Alcohol Use (last year)	OR (95% CI), p-value	OR (95% CI), p-value	OR (95% CI), p-value	OR (95% CI), p-value	OR (95% CI), p-value	OR (95% CI), p-value	OR (95% CI), p-value
Listwise (n = 2471)	1.225 (1.005–1.492), 0.044	1.100 (0.963–1.255), 0.159	1.644 (1.344–2.011), <0.001	1.019 (1.008–1.031), 0.001	5.533 (4.552–6.726), <0.001	1.299 (1.187–1.421), <0.001	0.907 (0.850–0.967), 0.003
ML-MAR (n = 4870)	1.193 (1.014–1.403), 0.033	1.053 (0.953–1.164), 0.313	1.631 (1.413–1.882), <0.001	1.017 (1.007–1.027), 0.001	4.914 (4.209–5.737), <0.001	1.299 (1.191–1.417), <0.001	0.900 (0.845–0.959), 0.001
MI-ML (n = 6391)	1.170 (0.986–1.388), 0.072	1.090 (1.004–1.183), 0.040	1.633 (1.367–1.951), <0.001	1.016 (1.004–1.027), 0.006	4.877 (4.132–5.758), <0.001	1.281 (1.180–1.391), <0.001	0.894 (0.827–0.965), 0.004
Alcohol Binge Drinking (last year)							
Listwise (n = 2459)	1.116 (0.906–1.375), 0.302	1.254 (1.099–1.430), 0.001	1.396 (1.120–1.739), 0.003	1.015 (1.003–1.027), 0.016	4.761 (3.531–6.420), <0.001	1.334 (1.210–1.471), <0.001	0.894 (0.840–0.952), <0.001
ML-MAR (n = 4842)	0.990 (0.831–1.179), 0.980	1.185 (1.077–1.304), 0.001	1.356 (1.146–1.605), <0.001	1.012 (1.001–1.022), 0.031	4.464 (3.565–5.588), <0.001	1.347 (1.229–1.477), <0.001	0.886 (0.835–0.939), <0.001
MI-ML (n = 6391)	0.967 (0.819–1.141), 0.690	1.166 (1.043–1.304), 0.007	1.369 (1.124–1.667), 0.002	1.013 (1.002–1.023), 0.016	4.281 (3.291–5.568), <0.001	1.392 (1.246–1.555), <0.001	0.883 (0.825–0.945), <0.001
Cigarette Use (last year)							
Listwise (n = 2457)	1.165 (0.744–1.826), 0.504	1.231 (0.945–1.603), 0.123	1.462 (1.095–1.952), 0.010	1.007 (0.989–1.025), 0.473	7.616 (4.110–14.111), <0.001	1.453 (1.278–1.651), <0.001	0.785 (0.714–0.889), <0.001
ML-MAR (n = 4846)	1.071 (0.741–1.548), 0.715	1.067 (0.890–1.279), 0.483	1.487 (1.159–1.908), 0.002	1.006 (0.989–1.023), 0.505	6.578 (4.032–10.732), <0.001	1.452 (1.287–1.639), <0.001	0.789 (0.717–0.869), <0.001
MI-ML (n = 6391)	1.004 (0.686–1.468), 0.985	1.039 (0.910–1.186), 0.569	1.682 (1.241–2.280), 0.001	1.002 (0.990–1.014), 0.714	5.700 (3.758–8.646), <0.001	1.814 (1.479–2.225), <0.001	0.691 (0.642–0.745), <0.001
Inhalant Use (last year)							
Listwise (n = 2463)	0.980 (0.714–1.277), 0.899	0.832 (0.692–1.000), 0.050	1.483 (1.157–1.901), 0.002	1.012 (0.994–1.030), 0.193	4.290 (3.005–6.126), <0.001	1.404 (1.236–1.594), <0.001	0.946 (0.853–1.049), 0.292
ML-MAR (n = 4849)	0.877 (0.667–1.153), 0.346	0.843 (0.722–0.985), 0.031	1.469 (1.163–1.855), 0.001	1.012 (0.998–1.026), 0.092	3.824 (2.932–4.986), <0.001	1.413 (1.250–1.597), <0.001	0.936 (0.848–1.034), 0.192
MI-ML (n = 6391)	0.835 (0.682–1.022), 0.081	0.832 (0.713–0.970), 0.019	1.551 (1.246–1.930), <0.001	1.017 (0.993–1.042), 0.155	3.846 (2.773–5.334), <0.001	1.564 (1.390–1.761), <0.001	0.902 (0.801–1.016), 0.090
Cannabis Use (last year)							
Listwise (n = 2451)	1.187 (0.760–1.855), 0.451	1.308 (1.012–1.691), 0.040	1.075 (0.767–1.507), 0.673	1.004 (0.988–1.020), 0.612	5.004 (2.141–11.692), <0.001	1.772 (1.556–2.017), <0.001	0.780 (0.702–0.867), <0.001
ML-MAR (n = 4847)	1.039 (0.724–1.491), 0.835	1.161 (0.986–1.367), 0.074	1.048 (0.842–1.303), 0.676	1.004 (0.992–1.016), 0.547	6.494 (3.502–12.049), <0.001	1.775 (1.554–2.026), <0.001	0.784 (0.707–0.869), <0.001
MI-ML (n = 6391)	0.989 (0.763–1.230), 0.934	1.086 (0.922–1.279), 0.321	1.200 (0.903–1.593), 0.208	0.998 (0.982–1.013), 0.766	4.927 (2.637–9.207), <0.001	2.569 (2.268–2.910), <0.001	0.677 (0.606–0.757), <0.001

SES: socio-economic class (SES) assessed using the ABEP scale.

The opposite was observed for negative beliefs about drugs. Negative beliefs at 9 months seemed to predict lower reporting of alcohol use, binge drinking, tobacco use and cannabis use at 21 months, after controlling for the same variables mentioned above. The strongest association occurred for cannabis use (OR = 0.78; 95% CI 0.70; 0.87).

Baseline drug use was also a predictor of drug use at 21 months, as expected. However, the strongest drug use predictor at baseline was tobacco smoking; individuals that reported tobacco smoking at baseline were almost 8 times more likely to also report tobacco smoking after 21 months (OR = 7.6; 95% CI 4.1; 14.1).

Except for cannabis use, being female was associated with past-year drug use at the 21-month follow up. Being female was associated with a 40% increase in past-year binge drinking (OR = 1.40; 95% CI 1.12; 1.74).

Considering that the direct effects were measured after taking into account the group allocation, all of the results presented here are independent of the #Tamojunto program. The odds ratios presented in the text refer to listwise analyses and are corroborated with missing data imputation (ML-MAR and MI-ML).

**Table 3** shows the two indirect effects (positive and negative beliefs) and the total indirect effect; indirect effects are reported in logit scale. We found a lack of significance regarding the indirect effects from the random assignment of the positive and negative beliefs, which in turn had lack of effect on the five dichotomous outcomes. Importantly, regardless of the methodological approach used to deal with missing data (listwise, ML-MAR, and MI-ML), the majority of the points estimated, and the confidence intervals were similar, indicating the stability of the estimations and findings. The results suggest that there is lack of effect of the program on the normative beliefs of the students or effect of the normative beliefs on past-year drug use. After Monte Carlo simulation (S3 Table), we observed that our sample size is robust to estimate the indirect effects properly, Details of power analysis are presented on Supplementary file (S3 Table).

**Table 3:** Mediation path.

Outcome: Alcohol Use (last year)	via Positive Beliefs	via Negative Beliefs	Total Indirect Effects
Listwise (n = 2471)	0.017 (-0.007 to 0.041), 0.160	0.004 (-0.010 to 0.017), 0.605	0.021 (-0.008 to 0.049), 0.154
ML-MAR (n = 4870)	0.006 (-0.015 to 0.026), 0.591	0.003 (-0.009 to 0.016), 0.602	0.009 (-0.018 to 0.036), 0.512
MI-ML (n = 6391)	0.005 (-0.014 to 0.024), 0.629	0.002 (-0.012 to 0.016), 0.742	0.007 (-0.016 to 0.030), 0.549
Outcome: Alcohol Binge Drinking (last year)			
Listwise (n = 2459)	0.019 (-0.008 to 0.046), 0.170	0.003 (-0.12 to 0.018), 0.706	0.022 (-0.010 to 0.054), 0.177
ML-MAR (n = 4842)	0.006 (-0.018 to 0.031), 0.604	0.003 (-0.011 to 0.018), 0.657	0.10 (-0.021 to 0.041), 0.420
MI-ML (n = 6391)	0.006 (-0.015 to 0.028), 0.564	0.004 (-0.010 to 0.018), 0.562	0.011 (-0.018 to 0.039), 0.690
Outcome: Cigarette Use (last year)			
Listwise (n = 2457)	0.025 (-0.013 to 0.063), 0.191	0.007 (-0.026 to 0.040), 0.672	0.032 (-0.019 to 0.083), 0.221
ML-MAR (n = 4846)	0.011 (-0.022 to 0.038), 0.520	0.007 (-0.020 to 0.034), 0.603	0.018 (-0.029 to 0.064), 0.455
MI-ML (n = 6391)	0.023 (-0.025 to 0.07), 0.355	0.014 (-0.017 to 0.045), 0.115	0.036 (-0.026 to 0.099), 0.254
Outcome: Inhalant Use (last year)			
Listwise (n = 2463)	0.022 (-0.012 to 0.056), 0.199	0.002 (-0.007 to 0.010), 0.675	0.024 (-0.012 to 0.060), 0.187
ML-MAR (n = 4849)	0.006 (-0.022 to 0.034), 0.688	0.002 (-0.006 to 0.010), 0.646	0.008 (-0.023 to 0.039), 0.626
MI-ML (n = 6391)	0.006 (-0.024 to 0.037), 0.301	0.004 (-0.006 to 0.014), 0.111	0.010 (-0.024 to 0.044), 0.258
Outcome: Cannabis Use (last year)			
Listwise (n = 2451)	0.035 (-0.020 to 0.090), 0.213	0.006 (-0.028 to 0.040), 0.730	0.041 (-0.026 to 0.107), 0.228
ML-MAR (n = 4847)	0.011 (-0.036 to 0.058), 0.645	0.005 (-0.024 to 0.033), 0.733	0.016 (-0.044 to 0.076), 0.605
MI-ML (n = 6391)	0.020 (-0.042 to 0.082), 0.180	0.007 (-0.027 to 0.041), 0.098	0.027 (-0.049 to 0.104), 0.172

## Discussion

The present study used a longitudinal design to test the hypothesis that the #Tamojunto prevention program would change normative beliefs about drug use at 9 months which in turns reduces drug use after 21 months. The null hypothesis, with indirect effects from the random assignment on drug use via positive and negative beliefs, was not rejected. Although the #Tamojunto program did not show statistically significant effects on reducing drug use [13], this paper intended to test whether the program affects normative beliefs and, in the long term, if these beliefs change drug use through a mediation process. However, #Tamojunto showed no success in changing normative beliefs as proposed by the theoretical model program [11,55].

This lack of mediated result is consistent with a systematic review of interventions using normative beliefs to prevent alcohol abuse among university students [56]. However, this finding contradicts the results found in the European *Unplugged* study, which showed reduced cigarette smoking, drunkenness episodes, and cannabis use through three common mediating factors: attitudes, refusal skills, and perception of the prevalence of the behavior among peers [5]. Other programs have also been successful in targeting normative beliefs in drug prevention programs to reduce drug use [57]: Project MYTRI [28], Project ALERT [30], All Stars [58], and the Aban Aya Youth Project [31].

It is important to state that the role of normative beliefs in prevention programs still remains controversial [59–61]. Two studies from the same group [62,63] found contradictory results among college students and their perceptions and attitudes towards alcohol [59]. Posterior analyses of the same samples found that interventions focusing on normative beliefs had variable efficacy, according to the characteristics of the communities where they were implemented [64]. Therefore, it is fair to infer that cultural and social characteristics play a role in the outcomes of such programs. This could partially explain the different findings from the Brazilian version of the program.

We must consider differences in the implementation process of *Unplugged* and #Tamojunto. In Brazil, the prevention program was delivered in schools as part of a public policy. Consequently, teacher participation was not voluntary, which may have compromised their engagement in the lessons, thus compromising the fidelity of the intervention, especially concerning normative beliefs [65]. Maintaining implementation standards is a difficult task in Brazil due to teachers’ poor pedagogical backgrounds and their beliefs and ideologies concerning drug use and public policies addressing this issue. This is important, as they are the facilitators of those prevention programs [66]. Data from an evaluation study of the implementation of #Tamojunto showed that only 57% of the classes in the program were completed as described in the manual due to a lack of time and proper knowledge of the content [67]. Additionally, the cultural adaptation of the program should also be considered, as important changes were made in the “Alcohol, Risk and Protection” lesson. Phrases that emphasized the importance of abstaining from alcohol use during adolescence were excluded and reflexive questions about how to avoid alcohol abuse and dependence were added [13], which might have caused changes in the target normative beliefs. Another important aspect that could explain the divergent results is the low quality of Brazilian public schools [68] and the high absenteeism rates of students [69]. These aspects can influence the learning process of students and therefore could be a possible explanation for the difficulty in understanding the activities that involve the changes in the normative beliefs proposed by #Tamojunto.

Despite these findings for mediation, this paper shows that there is a clear association between negative drug beliefs and lower consumption as well as between positive drug beliefs and higher consumption independent of the group allocation. These results show that investing in normative beliefs as mediators is valuable for reducing drug use, as there is a clear association, corroborating the international guidelines [7]. Programs that do not invest in changing normative beliefs do not show efficacy, even if they invested in enhancing self-esteem, psychological well-being and/or social competence, sports participation, or resistance skills [21,57,70]. Mediation analyses should be a research priority to help program developers understand how prevention programs are working and to provide information to modify the program, especially when it has negative impacts on substance use [29,71]. On the other hand, programs focusing on social influences do not seem to be beneficial to high-risk late adolescents. This may be because these subjects usually have already tried substances and using them is already part of their lifestyle choices and not a consequence of peer influence. In this case, normative beliefs play a less important role in the decision to use drugs [72].

A limitation of this study was the high number of students who were absent at baseline and/or at the follow up, resulting in an attrition rate of 37%. It is worth noting that according to a meta-analysis of school-based preventive interventions, attrition rates vary from 5 to 52% [73]. We also should report as a limitation of the study, that the normative beliefs were evaluated by a scale of perception of social acceptability about the use of drugs in general, without distinction of the type of drug used. Moreover, due to low understanding of the concept by adolescents [38], it was decided not to include in this study the question on beliefs about the number of friends who use drugs, as previously evaluated by Giannotta et al [5]. Another limitation is that normative beliefs are a complex construct that can be accessed via different scales in different studies, making comparison difficult, since there is no normative belief golden standard scale [5]. Moreover, we note that our study may suffer from confounding bias, since we cannot control for all the confounding factors that may affect the relationship between the predictor, the mediator, and the outcome.

The results of this study suggest that this program was not successful for changing normative beliefs, as proposed by the theoretical model of the program. The apparent inability of the program to impact mediators may be partially responsible for the negative outcomes previously identified. Negative normative beliefs seem to be a potential protective factor for drug use, while positive beliefs were identified as a potential risk factor for drug use; however, these beliefs were not influenced by the program itself. The activities of the program aimed at affecting normative beliefs should be improved. Consequently, the Brazilian version of the *Unplugged* program, #Tamojunto, needs significant revision to produce the intended effects, especially if it is to be delivered as a universal substance abuse prevention program.

## Supporting information

S1 Table CONSORT 2010 checklist of information to include when reporting a cluster randomised trial. (DOCX)

S2 Table Extension of CONSORT for abstracts 1, 2 to reports of cluster randomised trial. (DOCX)

S3 Table Monte Carlo Simulation for power for each outcome. (DOCX)

S1 Appendix Protocol. (PDF)

S2 Appendix Data set. (XLS)
