# Supplementary material for: The role of normative beliefs in the mediation of a school-based drug prevention program: A secondary analysis of the #Tamojunto cluster-randomized trial
Source: PLoS One. 2019 Jan 7;14(1):e0208072. doi: 10.1371/journal.pone.0208072 (PMC6322758; doi:10.1371/journal.pone.0208072)
Supplement: S3 Table — (DOCX) [file pone.0208072.s003.docx]

**S3 Table:** Monte Carlo Simulation for power for each outcome

Supplementary table 4 describes the results coming the Monte Carlo simulation 1,000 samples/replications. The *column Std. Dev.* is the standard deviation of the parameter estimates among all the samples/ replications. Just like the bootstrap method, the mean and standard deviation of the parameter estimates for a particular parameter over a large number of samples/ replications can be considered as the bootstrap estimates of that parameter and its standard error, respectively. The column S.E. Average stands for the average of standard error estimates over the samples/replications. The column M.S.E. represents the mean square error of each parameter, which equals the variance of the parameter estimates over all the samples/replications plus the squared bias. The column 95% Cover stands for coverage, representing the percentage of samples/ replications for which the 95% confidence interval covers the population parameter value. Major detail see Wang and Wang (2012).

Three criteria need to be examined in order to determine whether a sample size is sufficient (Muthén & Muthén, 2002) (1) The parameter and standard error biases should not exceed 10% for any parameter in the model; (2) the parameter and standard error biases for the focused parameter should not be larger than 5 %; and (3) the coverage should range from 0.91 to 0.98. The percentage of bias in the parameter estimate of interest (i.e., indirect effects) is calculated by subtracting its population value from its average parameter estimate, and then dividing by its population. 5 out 10 indirect effect parameters showed to be below than 10%. The percentage of bias of standard error for the parameter of interest was calculated by subtracting the standard deviation of the parameter estimate from the average of the estimated standard error, and then dividing by the standard deviation. Regarding this criterion, all our parameters showed to be much less than the cut-off point of 5% (column called criterion 2). Lastly, regarding than coverage, column criterion 3, all our parameters showed to be between 91 and 98%. This indicates that our sample size is robust to estimate the indirect effects properly.

**References**

**Muthén, L. K., & Muthén, B. O. (2002). How to use a Monte Carlo study to decide on sample size and determine power. *Structural equation modeling, 9*(4), 599-620.**

**Wang, J., & Wang, X. (2012). *Structural equation modeling: Applications using Mplus*: John Wiley & Sons.**
