# Supplementary material for: The role of normative beliefs in the mediation of a school-based drug prevention program: A secondary analysis of the #Tamojunto cluster-randomized trial
Source: PLoS One. 2019 Jan 7;14(1):e0208072. doi: 10.1371/journal.pone.0208072 (PMC6322758; doi:10.1371/journal.pone.0208072)
Supplement: S1 Appendix — (PDF) [file pone.0208072.s004.pdf]

REGISTRO BRASILEIRO DE Ensaios Clínicos

USUÁRIO SENHA ENTRAR Esqueceu a senha? Registrar-se

PT | ES | EN

NOTÍCIAS | SOBRE | AJUDA | CONTATO

Buscar ensaios

BUSCA AVANÇADA

HOME / ENSAIOS REGISTRADOS /

RBR-4mnv5g
Avaliação do processo de implantação de programas de prevenção ao uso de drogas para crianças e adolescentes em ambiente escolar (Ensaio randomizado controlado)

Data de registro: 3 de Julho de 2014 às 16:30

Last Update: 6 de Fev. de 2015 às 15:26

Tipo do estudo:

Intervenções

Título científico:

PT-BR	EN
Avaliação do processo de implantação de programas de prevenção ao uso de drogas para crianças e adolescentes em ambiente escolar (Ensaio randomizado controlado)	Evaluation of the implementation of school prevention program s to drug use for children and adolescents (Randomized controlled trial)

Identificação do ensaio

Número do UTN: U1111-1158-8901

Título público:

PT-BR	EN
Ensaio Controlado Randomizado de Programa Escolar de Prevenção para uso de drogas Unplugged (#Tamojunto) entre os adolescentes brasileiros	Randomized Controlled Trial of the School Prevention Program for drug Unplugged (#Tamojunto) among Brazilian adolescents

Acrônimo científico:

Acrônimo público:

Identificadores secundários:

19944213.5.0000.5505
Órgão emissor: Plataforma Brasil

473.498
Órgão emissor: Comitê de Ética em Pesquisa da Universidade Federal de São Paulo

Patrocinadores

Patrocinador primário: Universidade Federal de São Paulo - UNIFESP

Patrocinadores secundários:

Instituição: Ministério da Saúde

Instituição: Universidade Federal de São Paulo - UNIFESP

Fontes de apoio financeiro ou material:

Instituição: Centro Brasileiro de Informações sobre Drogas Psicotrópicas - CEBRID

Instituição: Ministério da Saúde

Condições de saúde

Condições de saúde ou problemas:

PT-BR

Transtornos mentais e comportamentais devidos ao uso de álcool , Auto-intoxicação por e exposição, intencional, a narcóticos e psicodislépticos [alucinógenos]

EN

Mental and behavioral disorders due to use of alcohol, self-poisoning by and exposure, intentional, to narcotics and psychodysleptics [hallucinogens]

Descritores gerais para as condições de saúde:

PT-BR

F00-F99: V - Transtornos mentais e comportamentais

EN

F00-F99: V - Mental, behavioural disorders

Descritores específicos para as condições de saúde:

PT-BR

F10.1: Transtornos mentais e comportamentais devidos ao uso de álcool - uso nocivo para a saúde

ES

F10.1: Trastornos mentales y del comportamiento debidos al uso de alcohol, uso nocivo

EN

F10.1: Mental and behavioural disorders due to use of alcohol, harmful use

PT-BR

X62: Auto-intoxicação por e exposição, intencional, a narcóticos e psicodislépticos [alucinógenos] não classificados em outra parte

ES

X62: Envenenamiento autoinfligido intencionalmente por, y exposición a narcóticos y psicodislépticos (alucinógenos), no clasificados en otra parte

EN

X62: Intentional self-poisoning by and exposure to narcotics and psychodysleptics [hallucinogens], not elsewhere classified

Intervenções

Categorias das intervenções

Behavioural

Intervenções:

PT-BR

Grupo experimental: Com 6469 participantes, a intervenção consiste na aplicação do programa #Tamojunto (guiado por manual) em sala de aula, pelo professor de cada turma, treinado pelo Ministério da Saúde. O programa escolar trabalha o desenvolvimento de habilidades para a vida para a prevenção ao uso de drogas, que compreende: o desenvolvimento de pensamento crítico, tomada de decisões, solução de problemas, pensamento criativo, comunicação eficaz, habilidade

EN

Experimental group: With 6469 participants, the intervention consists of the implementation of #Tamojunto program (guided by a manual) by the teacher responsible for the classroom. Teachers will be trained by the Ministry of Health. The goal of *tamojunto program is to implement life skills training to prevent drug use through developing “critical thinking , “decision making” , “problem solving” , “creative thinking” , “effective communication” , “interpersonal skills” , “self-awareness” , “empathy” and “dealing

interpessoal, auto percepção, empatia e lidar com emoções. Soma-se a estas habilidades, as crenças normativas, onde há a reavaliação das expectativas normativas, desenvolvendo reflexões sobre a prevalência e aceitabilidade do uso de drogas. O programa é dividido em 12 aulas (Abertura do Unplugged / Fazer parte ou não de um grupo / Escolhas – Álcool, risco e proteção / Suas crenças, normas e informações refletem a realidade? / Fumando a droga cigarro – Informe-se / Expresse-se / Atenção no mundo e em sua vida / Novo no pedaço / Drogas – Informe-se / Estratégias de enfrentamento / Solução de problemas e tomadas de decisões / Estabelecimento de metas). Os dados são coletados em 2 fases (fase I e II), onde os resultados são comparados para analisar se houve diferença no consumo de álcool e outras drogas após a intervenção

Grupo controle: Com 6457 participantes, o grupo controle não recebeu nenhuma intervenção em sala de aula e apenas responderá aos mesmos questionários do grupo experimental sobre o consumo de drogas, em dois momentos

with emotions". Besides to the life skills training it is expected that the student can evaluate "normative beliefs", developing new insights about the prevalence and acceptability of drug use. The program is divided in 12 classes : Opening of the Unplugged / Being a part of a group or not? / Choices – Alcohol: risk and protection / Do your beliefs, choices and information reflect the reality? / Smoking cigarette - Enquire / Express Yourself / Attention in the world and in your life / Party tiger / Drugs - Enquire / Coping strategies / Problems Solving and decision making / Goals setting). The data are collected in two phases -Phase I and II - and the results are compared to evaluate if there are differences considering the use of alcohol and other drugs after the intervention

Control group: With 6457 participants, the control group didn't receive the intervention but will answer to the same questionnaire of the experimental group about the use of alcohol and other drugs in two different moments

Descritores para as intervenções:

PT-BR

I02.903.573: Instrução Programada como Assunto

ES

I02.903.573: Instrucción Programada como Asunto

PT-BR

F02.784.629.272: Orientação Infantil

ES

F02.784.629.272: Orientación Infantil

Recrutamento

Situação de recrutamento: Recruitment completed

País de recrutamento

Brazil

Data prevista do primeiro recrutamento: 2014-08-18

Data prevista do último recrutamento: 2014-12-08

Tamanho da amostra alvo:	Gênero para inclusão:	Idade mínima para inclusão:	Idade máxima para inclusão:
12926	-	13 Y	16 Y

Critérios de inclusão:

PT-BR

Ser aluno matriculado no 8º ano (antiga 7ª série) de uma das escolas públicas selecionadas randomicamente a partir da

EN

Being a student enrolled in grade 8 (old 7th grade) of one of the public schools randomly selected from the list of INEP, in

lista do INEP, nas cidades de São Paulo, Florianópolis, Fortaleza, Tubarão, São Bernardo do Campo, Brasília e consentir responder ao questionário de pré e pós teste

São Paulo, Florianopolis, Fortaleza, Tubarão, São Bernardo do Campo, Brasilia and consent to respond to the pre and post-test questionnaire

Critérios de exclusão:

PT-BR
Questionários sem o preenchimento adequado dos códigos; Alunos com déficit cognitivo; Recusa no preenchimento do questionário

EN
Questionnaires without adequate codes; Students with cognitive impairment; Refusal in completing the questionnaire

Tipo do estudo

Desenho do estudo:

PT-BR
Ensaio clínico de prevenção randomizado controlado, paralelo, com 2 braços, aberto, prospectivo

EN
Prevention trial randomized controlled, parallel, 2-arm, open, prospective

Programa de acesso expandido	Enfoque do estudo	Desenho da intervenção	Número de braços	Tipo de mascaramento	Tipo de alocação	Fase do estudo
Nenhum	Prevention	Parallel	2	Abrir	Randomized-controlled	N/A

Desfechos

Desfechos primários:

PT-BR
Desfecho esperado: 1) Avaliar o uso no ano (pelo menos um uso nos últimos 12 meses) das seguintes drogas: álcool, tabaco, maconha, inalantes e cocaína - 2) Avaliação realizada pelo questionário EU-DAP de auto-preenchimento para detecção de consumo de drogas entre adolescentes - 3) Constatação de uma variação de pelo menos 5% nas medições pré e pós na comparação do grupo experimental e controle

EN
expected Outcome: 1) To evaluate the use of the year (at least one use in the last 12 months) of the following drugs: alcohol, tobacco, marijuana, inhalants and cocaine - 2) Evaluation by the US-DAP questionnaire self-completion for drug use detection among adolescents - 3) detection of a range of at least 5% in pre and post measurements comparing the experimental and control groups

Desfechos secundários:

PT-BR
Não foram encontrados desfechos secundários

EN
No secondary outcomes were found

Contatos

Contatos para questões públicas

Nome completo: Joselaine Ida da Cruz

Endereço: Rua Botucatu, 740 - 4. andar

Cidade: São Paulo / Brazil

CEP: 04023-900

Fone: +55(11)94872 5475

E-mail: jjosi.cruz@gmail.com

Filiação: Universidade Federal de São Paulo - UNIFESP

Contatos para questões científicas

Nome completo: Zila van der Meer Sanchez

Endereço: Rua Botucatu, 740 - 4. andar

Cidade: São Paulo / Brazil

CEP: 04023-900

Fone: +55(11)98934 8282

E-mail: zila.sanchez@gmail.com

Filiação: Universidade Federal de São Paulo - UNIFESP

Contatos para informação sobre os centros de pesquisa

Nome completo: Zila van der Meer Sanchez

Endereço: Rua Botucatu, 740 - 4. andar

Cidade: São Paulo / Brazil

CEP: 04023-900

Fone: +55(11)98934 8282

E-mail: zila.sanchez@gmail.com

Filiação: Universidade Federal de São Paulo - UNIFESP

Links adicionais:

Download no formato ICTRP

Download no formato XML OpenTrials

OpenTrials v1.2
